# A survey-based evaluation of ambulance staff awareness of vitamin D and risk of deficiency in a UK ambulance service

**DOI:** 10.29045/14784726.2021.9.6.2.40

**Published:** 2021-09-01

**Authors:** Larissa Stella Prothero, Theresa Foster

**Affiliations:** East of England Ambulance Service NHS Trust ORCID iD: https://orcid.org/0000-0002-5440-8429; East of England Ambulance Service NHS Trust

**Keywords:** ambulances, deficiency, emergency services, vitamin D

## Abstract

**Background:**

Healthcare professions, shift-working and indoor-working are risk factors for vitamin D deficiency. The aim of this survey was to investigate ambulance staff awareness of vitamin D, and the risks associated with deficiency, to inform the need for appropriate well-being resources.

**Methods:**

A purpose-designed, 20-question survey was developed, based on a validated vitamin D questionnaire, to explore staff vitamin D knowledge and self-health in one UK ambulance service. Disseminated during June/July 2020, survey completion was voluntary, and responses obtained were analysed using descriptive and thematic approaches.

**Results:**

A total of 384 survey responses were received; 41% (n = 156) of respondents were male. Over half worked within emergency operational service delivery (57%; n = 219). Respondents were predominantly ‘White British’ (92%; n = 352). According to the Fitzpatrick Scale, most described themselves as having a ‘Medium, between white to moderate brown: sometimes mild burns, gradual tan’ complexion (47%; n = 182). The majority felt they got sufficient sunlight exposure when at home (66%; n = 253), but not at work (58%; n = 222). Almost one fifth (17%; n = 66) had received a diagnosis of vitamin D deficiency. Forty percent took vitamin D supplements: 12% (n = 45) as advised by a medical professional; 28% (n = 107) self-directed to prevent deficiency. The ability of respondents to recognise known factors that affect vitamin D production in the skin, good vitamin D food sources and individuals at risk of vitamin D deficiency were variable. Respondents commented on their lack of vitamin D awareness, vitamin supplementation, COVID-19, work arrangements and access to sunlight.

**Conclusions:**

Ambulance staff are at risk of vitamin D deficiency irrespective of their role: vitamin D awareness, access to sunlight and use of vitamin D supplements are variable. For affected individuals, the impact of vitamin D deficiency can be significant, requiring absence from work. The development of appropriate vitamin D and well-being resources appears to be warranted.

## Introduction

Vitamin D is primarily produced by the skin on exposure to ultraviolet B sunlight but can also be obtained from the diet. It has varied and important roles in the human body, in particular in bone, muscle and immune system function (Holick, 2019). Vitamin D deficiency is considered to be a global public health problem (Palacios and Gonzalez, 2014) and is common in the UK. It has been reported that up to a quarter of the population has vitamin D deficiency (Bates et al., 2014), which contributed to the recent Scientific Advisory Committee on Nutrition (SACN) guidelines to address this health concern (SACN, 2016).

It is now recognised that certain factors are linked to vitamin D deficiency: low sunlight exposure, dark skin and low vitamin D consumption. Common signs of vitamin D deficiency include increased infections and risk of illness; fatigue and tiredness; bone, back or muscle pain; depression; low bone mineral density (Holick, 2019).

Recently, occupation has been recognised as a factor linked to increased risk of vitamin D deficiency: shift-working, indoor-working and healthcare professions have been associated with a higher risk of vitamin D deficiency (Alefishat and Abu Farha, 2016; Divakar et al., 2019; Fajardo et al., 2019; Sowah et al., 2017).

To date, no literature has been found that specifically explores the incidence of vitamin D deficiency in the ambulance workforce. This despite the ambulance service comprising day workers, night workers, rotating shift workers, individuals that work solely indoors and others that work outdoors. However, anecdotal evidence has revealed vitamin D deficiency to be an issue for emergency ambulance staff. Consequently, the aim of this survey was to explore staff awareness of vitamin D and the risks associated with deficiency, to inform the development of appropriate well-being support and resources.

## Methods

An online, Microsoft Forms-based 20-question survey (available on request) was purpose-designed by the authors, based on a validated vitamin D questionnaire (Hourihane et al., 2019; O’Connor et al., 2018). The survey was advertised via a service e-newsletter across the East of England Ambulance Service NHS Trust (EEAST) between 16 June 2020 and 12 July 2020 to obtain a convenience sample. Survey completion was voluntary and anonymous, and informed consent was obtained prior to completion. No personally identifiable information was collected, and all responses were securely stored electronically (password-protected). Question topics included service role and working hours, sufficiency of sunlight exposure, factors affecting vitamin D production, dietary sources of vitamin D, risk factors for vitamin D deficiency and vitamin D deficiency diagnosis and management.

Descriptive statistical techniques were used to analyse responses. The final survey question was a free-text option for additional comments to support any responses given. These were thematically analysed, grouping responses with the same meaning together into themes. All questions were mandatory (except for the final free-text box) to ensure a 100% survey completion rate.

## Results

### Respondent demographics

A total of 384 surveys were received from both male (41%, n = 156) and female (59%, n = 227) respondents, representing approximately 9% of EEAST staff. All age categories from 20 years were represented (20–29 years: 13%, n = 49; 30–39 years: 21%, n = 82; 40–49 years: 31%, n = 118; 50–59 years: 28%, n = 108; 60 years or more: 7%, n = 27). Fourteen percent of respondents (n = 55) reported they were experiencing either the menopause or andropause, and of the female respondents, 3.5% (n = 8) reported they were either pregnant, breast-feeding or both.

Most survey responses were obtained from emergency operational delivery staff (57%, n = 219), however all key service functions were represented (service support: 18%, n = 68; ambulance operations centres: 16%, n = 62; scheduled patient transport: 5%, n = 18; resilience and special operations: 3%, n = 11; preferred not to say: 1%, n = 6). Typically, respondents worked ‘days and nights’ (37%, n = 140) or ‘days only’ (30%, n = 114), with 16% working ‘days and lates’ (n = 61) and 15% working combinations of ‘days, lates and nights’ shifts (n = 58). In this service, the term ‘lates’ is used for shifts starting after 09:00 and scheduled to finish before 00:00. Eleven respondents (3%) worked either night shifts only, or ‘lates and nights’. Respondents predominantly worked full-time hours (i.e. 30+ hours per week; 85%, n = 325), with the remainder working fewer than 30 hours per week.

When asked to describe their ethnicity, most respondents reported to be ‘White British’ (92%, n = 352), however other ethnic groups (White Irish, White/Asian, White/Black African or Caribbean, White Other, Indian, Black British, Black African or Caribbean and Chinese) were represented.

**Figure 1:**
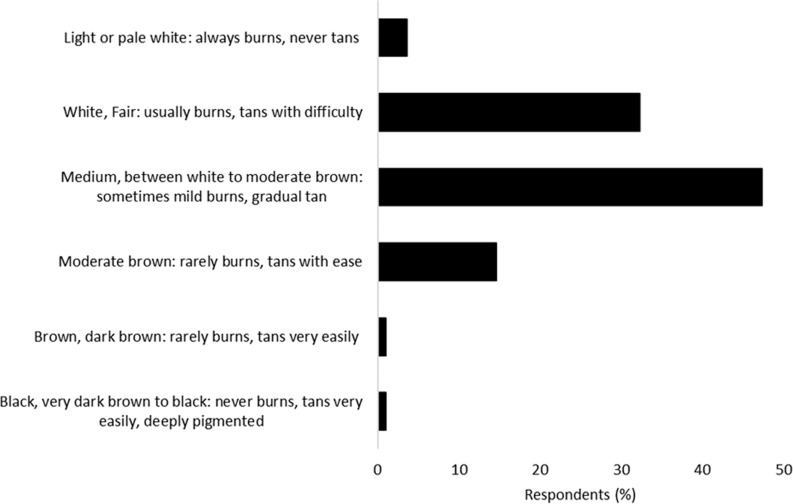
Figure 1. Fitzpatrick Scale profile of survey respondents.

The Fitzpatrick Scale is commonly used to classify a person’s complexion in relation to their tolerance to sunlight (Fitzpatrick, 1988; Roberts, 2009). The majority of staff described themselves as having either ‘Medium, between white to moderate brown: sometimes mild burns, gradual tan’ (47%; n = 182) or ‘White, Fair: usually burns, tans with difficulty’ (32%, n = 12; Figure 1).

### Vitamin D awareness

**Figure 2:**
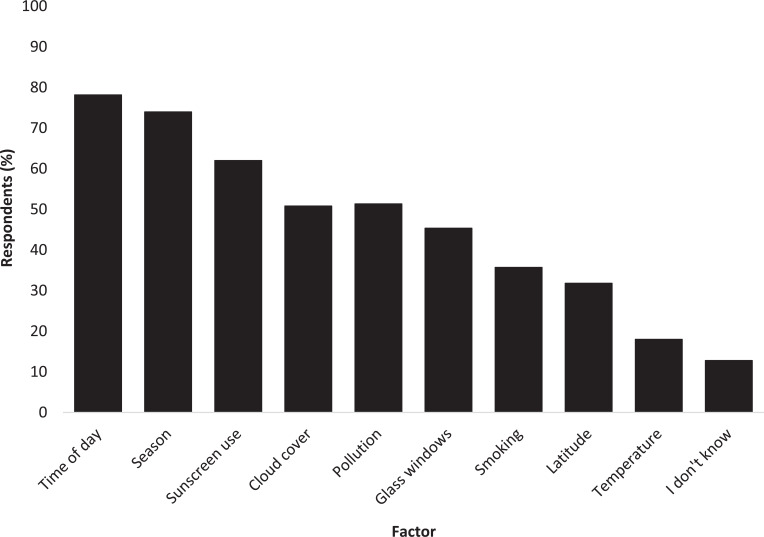
Figure 2. Recognition of known factors to affect vitamin D production.

To understand knowledge regarding factors affecting vitamin D production in the skin, respondents were presented with a list of nine factors known to reduce vitamin D production to varying extents and asked to identify those they knew. The three most recognised were ‘time of day’ (78%, n = 300), ‘season’ (74%, n = 284) and ‘sunscreen use’ (62%, n = 238; Figure 2).

**Figure 3:**
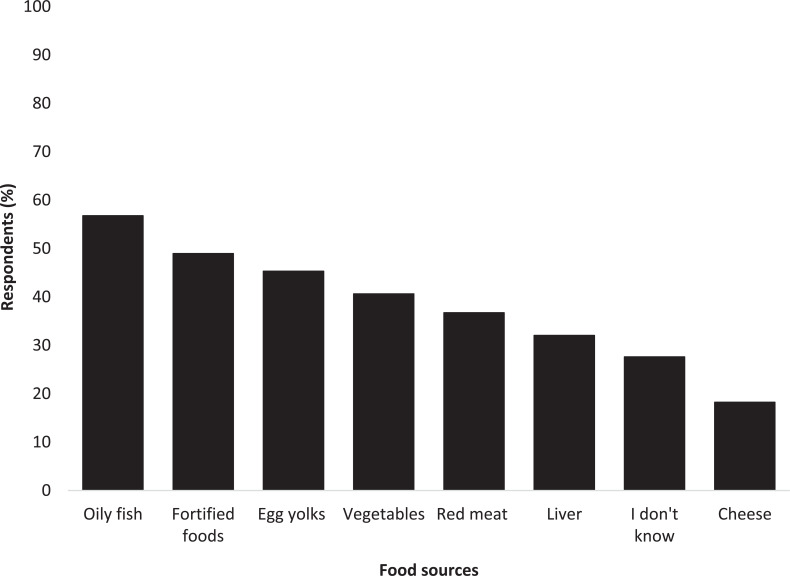
Figure 3. Recognition of foods known to be good sources of vitamin D (Note: ‘Fortified foods’ includes fat spreads and some breakfast cereals).

Knowledge of good vitamin D food sources was variable (Figure 3). All foods listed, except for vegetables, are considered to be good sources of vitamin D, alongside that which is produced in the skin. The three most recognised were ‘oily fish’ (57%, n = 218), ‘fortified foods’ (49%, n = 188) and ‘egg yolks’ (45%, n = 174). Over 40% (n = 156) of respondents wrongly considered vegetables to be a good vitamin D food source.

**Figure 4:**
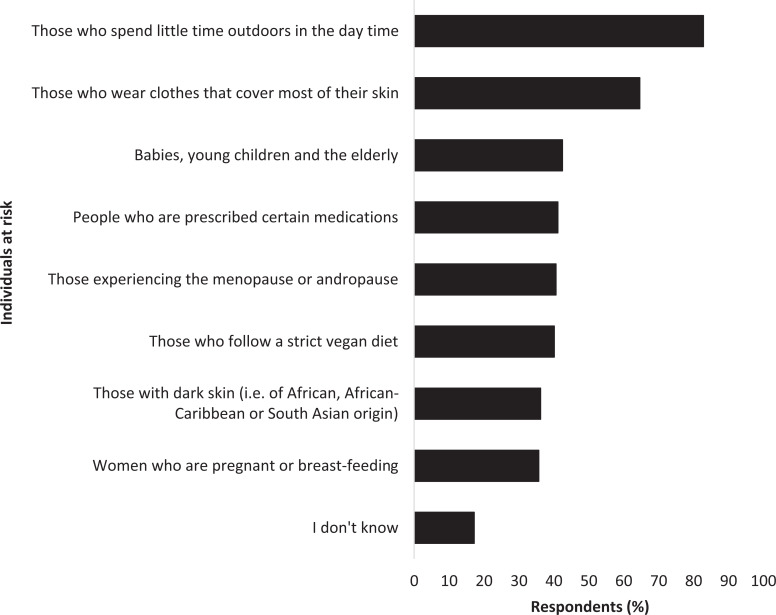
Figure 4. Recognition of individuals at risk of vitamin D deficiency (Note: ‘Those who spend little time outdoors in the day’ includes nightshift workers and care home residents; ‘prescribed certain medications’ includes steroids, weight-loss drugs and anti-convulsants).

The ability of respondents to recognise individuals at risk of vitamin D deficiency was also variable (Figure 4). All examples given were individuals at risk of deficiency. ‘Those who spend little time outdoors in the day time’ (83%, n = 318) and ‘Those who wear clothes that cover most of their skin’ (65%, n = 248) were correctly identified by most respondents.

### Vitamin D deficiency

When asked about levels of agreement with the statement ‘I feel I get sufficient sunlight exposure when at work’, most respondents either disagreed or strongly disagreed (58%, n = 222); in contrast, they felt they got sufficient sunlight exposure when at home (66%, n = 253). When respondents were asked if they were concerned about having vitamin D deficiency, responses ranged from ‘very unconcerned’ to ‘very concerned’, with most stating they were ‘neither unconcerned or concerned’ (35%, n = 135) or ‘concerned’ (33%, n = 128).

**Figure 5:**
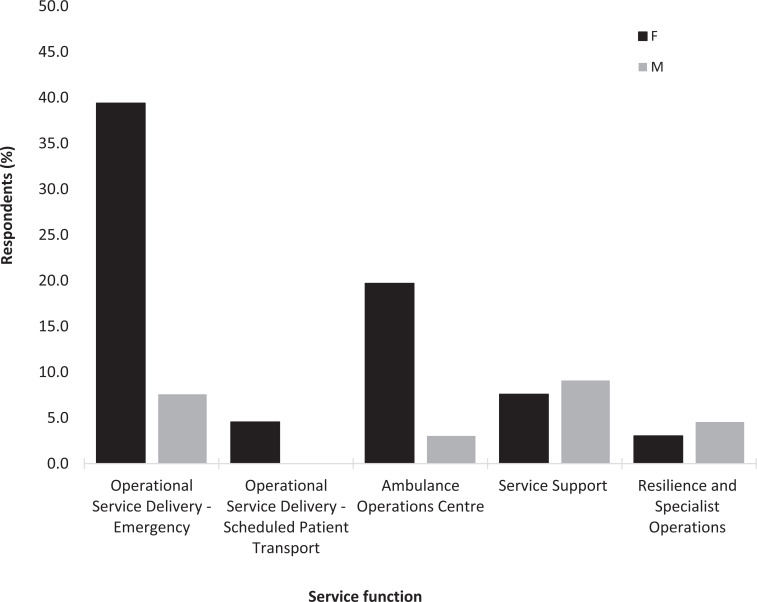
Figure 5. Incidence of vitamin D deficiency according to service function (subgroup n = 66; F = female, M = male. Note: Service Support roles include Finance, Clinical Audit, Education and Training and Human Resources etc; Resilience and Specialist Operations includes Hazardous Area Response Team and the Air Ambulance).

Almost one in five respondents (17%, n = 66) reported to have been told they had vitamin D deficiency by a medical professional. Of this subgroup, approximately three quarters were female (76%, n = 50), and all age ranges and service functions (Figure 5) were represented.

**Figure 6:**
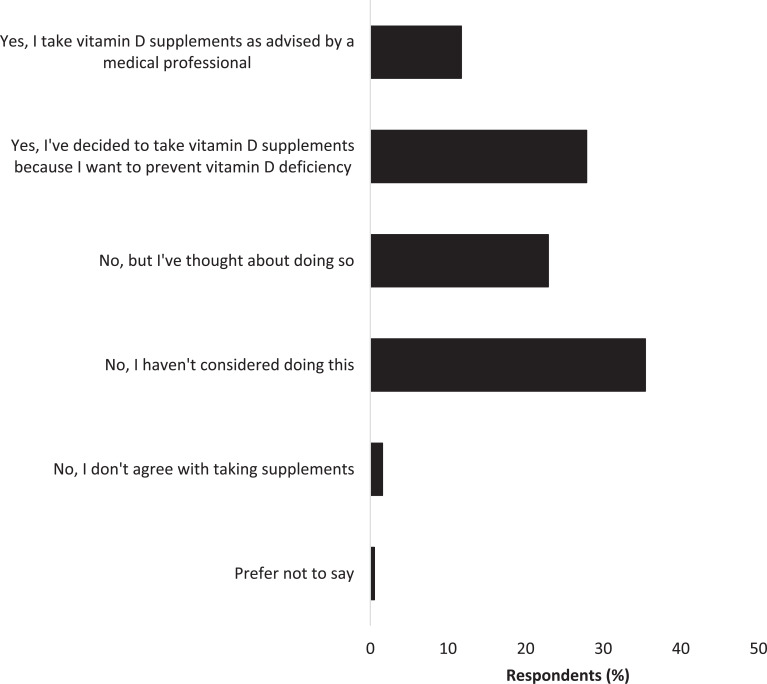
Figure 6. Respondent views on vitamin D supplementation.

Forty percent of all respondents took vitamin D supplements: 12% (n = 45) as advised by a medical professional, and 28% (n = 107) self-directed to prevent deficiency. However, the majority of respondents (35%, n = 136) had not considered taking such dietary supplements (Figure 6).

Exploring staff interest in vitamin D and self-health more widely, almost all respondents (93%, n = 358) stated they would consider having a capillary blood vitamin D assessment if it were made available (‘not sure’: 4%, n = 16; ‘no’: 3%, n = 10), and more than half (56%; n = 216) scored 6 or more on a scale of 0 = very unlikely to 10 = very likely, indicating they were now likely to make lifestyle changes (however small or large) to reduce their risk of vitamin D deficiency.

### Respondents’ supporting comments

Of the 384 respondents, 68 (18%) provided free-text comments at the end of the survey. Thematic analysis revealed nine key themes, which are presented below in order of decreasing occurrence.

#### Lack of awareness

Most frequently, respondent comments highlighted the general lack of awareness regarding vitamin D deficiency, but indicated a genuine interest in improved vitamin D knowledge:

*I have no real awareness of vitamin D deficiency.* (R289)

*I had not thought about vitamin D deficiency, I would like to get tested if needed and would consider to taking it if I had low levels.* (R354)

#### Deficiency impact

The next most frequent theme encompassed the potential impact of vitamin D deficiency and its severity, with respondents describing their own experiences:

*It took a long time and several return visits to GP with muscle weakness joint pain type symptoms before I had a blood test which revealed I was vit D deficient … I wish I had known sooner because I have suffered with these and other symptoms including low mood and mood swings etc … This has all impacted my life adversely only now beginning to get back on track (I hope).* (R84)

*… [My GP] prescribed it for an irregular heartbeat.* (R154)

*I fractured my wrist 3 years ago and following a dexa scan was found to be osteopenic …* (R320)

#### Dietary supplements

The third most common theme related specifically to vitamin D supplementation. While some respondents took vitamin D alone, others took it as part of a multivitamin supplement:

*I have been taking a Vitamin D supplement since Feb 2020.* (R104)

*Normally take multivitamins daily which includes vit D.* (R277)

One respondent raised their concerns relating to the expense of vitamin D supplements (R262), while another raised the issue of availability of vegan-friendly (lanolin-free) vitamin D preparations (R293). In addition, another respondent queried vitamin D dosing, and whether vitamin D should be taken with vitamin K2 (R318).

#### COVID-19

The survey was designed prior to the COVID-19 pandemic, so not intended to capture views specifically relating to it; however, the next most frequent comments were related to COVID-19. Some respondents identified a potential issue with the requirements of wearing personal protective equipment (PPE), while others reported they had commenced or increased vitamin D supplement intake:

*Since covid, myself and my family have been taking vit d supplements.* (R324)

*I have recently upped my vitamin D intake due to my own research and present covid threat.* (R319)

In addition, one respondent reported apparent vitamin D benefits when becoming unwell during COVID-19:

*I had suspected [COVID-19] in Feb & GP believes the respiratory element was minimised possibly because I take Vit D3 ... and advised me to keep taking it. I have felt its helped prevent any virus getting into my system and taking hold. I dont know if it was [COVID-19]. I was ill, but no cough, no severe breathing problems. Im waiting for an antibody test.* (R145)

#### Work arrangements

In this theme, respondents shared the difficulties of their work arrangements, being outdoors and having access to sunlight:

*I work in an office in the middle of the building which has no natural light. I work during daylight hours and especially during the shorter days of the year I potentially get no access to any daylight at all.* (R362)

*I was vitamin D deficient until I gave up night shifts. My bloods are now within normal parameters 3 months later.* (R164)

#### Sunlight exposure

Related to the previous theme, respondents’ comments highlighted that exposure to sunlight is not necessarily difficult for some respondents, while for others pre-existing medical issues can make it challenging:

*I am always outside when home, throughout year.* (R246)

*I suffer from reaction to the sun ? [Polymorphic Light Eruption] so have to avoid sunlight as much as possible.* (R61)

*having suffered from basal cell carcinomas in the past I have to limit the amount of time I spend in the sun and if in the sun for any length of time use a factor 50 suncream.* (R380)

#### Deficiency risk factors

Respondents commented on various known risk factors for vitamin D deficiency. The most frequently mentioned were the menopause and post-menopause:

*I have been supplementing my nutrition with vit D, calcium and a Magnesium combo which helps with the symptoms of menopause and thyroid problems (I fall into both categories) for nearly 2 years it’s also been good for my joints.* (R129)

*I began taking strong dose vitamin D supplements on surgical menopause as I believed this would help my symptoms.* (R363)

Seasonality, appropriate diet, underlying health concerns and pregnancy were also commented on, to a lesser extent:

*I only take vitamin D supplements from September/October to March/April.* (R142)

*I know I am at risk because I am a pescatarian … So I eat a lot of brown mushrooms and try to eat more oily fish.* (R72)

*Having had breast cancer I do worry about the amount of Vitamin D.* (R265)

*I had Vit D deficiency after having my first child.* (R178)

#### Self-learning

The penultimate theme captured comments relating to respondent self-directed learning and well-being. It appeared that such learning initiated and supported changes to improve overall health:

*I have underactive thyroid and as part of the research I did when diagnosed decided to start taking vitamin D, which I have for the past 3 years. I've noticed a significant reduction in catching cold/flu illnesses.* (R202)

*I recently saw [National Institute for Health and Care Excellence] advice that most adults in the UK should take vit D supplements and have thought about doing so.* (R350)

#### General well-being

The final theme included generic comments relating to vitamin D supplements, the impacts of vitamin D deficiency and service delivery:

*I’m surprised that more people are not taking Vit D supplements as NHS recommends low level supplements for pretty much everyone especially in the winter months …* (R45)

*… reduced Vit D levels in young women can result in less grip strength which can affect carrying capability …* (R189)

*Considering [muscular-skeletal] related injuries are high in Trust, it would be interesting to see the correlation between Vitamin D deficiency and staff absences.* (R24)

## Discussion

It is understood that this is the first study of vitamin D awareness and deficiency risk involving ambulance personnel. The results indicated the respondent cohort generally represented the staff demographic in terms of sex, age and area of work across all key functions of the service. Most respondents described themselves as ‘White British’ with a medium to white complexion (although various ethnic minority groups and those with darker skin were represented). Similarly, most respondents reported to work full-time hours, working both day and night shifts or ‘days only’.

From the responses provided, there appeared to be a genuine interest for improved understanding of vitamin D and self-health. Responses indicated varied and lacking knowledge relating to factors affecting vitamin D production, good vitamin D food sources and vitamin D deficiency risk factors; however, the need for sun exposure was recognised by most. While it is not known exactly how much time is needed in the sun to make sufficient vitamin D to meet the body’s requirements and prevent deficiency (NICE, 2018a), respondents generally felt they got sufficient sunlight exposure at home, but not at work. A number of free-text comments highlighted the difficulties some staff faced getting sufficient sunlight – night shifts, particularly during winter months, limited access to natural light; prolonged use of PPE items (particularly during COVID-19); and pre-existing medical conditions that impacted ability to access sunlight.

Approximately one in five of all respondents had been diagnosed with vitamin D deficiency, and it was made apparent that the impact of this (physical and mental) could be significant, requiring absence from work. Since both sexes, all ages and key service functions were represented in the vitamin D deficient subgroup, it appears all ambulance staff are at risk of vitamin D deficiency.

National Institute for Health and Care Excellence (NICE, 2018b) guidance advises all adults living in the UK to take a daily supplement containing 400 international units (10 micrograms) of vitamin D throughout the year, especially in the winter months. However, only 40% of survey respondents reported to take vitamin D supplements, and of those not taking any, most had not considered doing so. It appears staff require additional information about vitamin D preparations, and which are the most beneficial for their health, e.g. a vitamin D supplement versus a multivitamin. In addition, some menopausal/post-menopausal women did report the benefits of using a multiple vitamin supplement approach (to include vitamin D) to aid their symptom management.

It should be noted that routine staff screening for vitamin D deficiency is not currently recommended. According to NICE, testing is warranted for individuals with symptoms of osteomalacia, osteoporosis, chronic widespread pain, hypocalcaemia, Paget’s disease and falls and prior to treatment to correct vitamin D deficiency (NICE, 2018b).

The following limitations are acknowledged: while a large number of staff participated in this survey, it is recognised that this is a single service evaluation so the generalisability of the findings may be limited; owing to the range of risk factors that contribute to vitamin D deficiency, variation in prevalence at service level may exist; the purpose-designed survey used has yet to be formally validated, although it is based on a validated questionnaire designed for the UK public; and as an anonymous survey, multiple entries from a single respondent could not be identified. It should also be noted that this survey was conducted during the COVID-19 pandemic, therefore use of additional PPE and lockdown conditions may have impacted findings.

## Conclusion

There is scope for improved awareness of vitamin D and the risks for deficiency in the ambulance setting. It appears service staff are at risk of vitamin D deficiency irrespective of their role: access to sunlight and use of vitamin D supplements are variable. The physical and mental impacts of deficiency can be significant, which can require absence from work. The development of appropriate vitamin D and well-being resources appears to be warranted.

## Author contributions

The study concept and survey were devised by LP. Both LP and TF have contributed to the data analysis and reporting of findings. SQUIRE guidelines and the CHERRIES checklist provided frameworks for the preparation of this manuscript (Eysenbach, 2004; Ogrinc et al., 2015). LP acts as the guarantor for this article.

## Conflict of interest

Primary author (LP) is a member of the British Paramedic Journal Editorial Group.

## Ethics

The Health Research Authority (HRA) (http://www.hra-decisiontools.org.uk/research/) did not deem the study to be research, therefore HRA approval was not sought. EEAST NHS Trust Clinical Best Practice Group has approved this study.

## Funding

None.

## References

[bibr_1] Alefishet E. & Abu Farha R. (2016). Determinants of Vitamin D status among Jordanian employees: Focus on the night shift effect. *International Journal of Occupational Medicine and Environmental Health*, 29(5), 859–870. 27518893 10.13075/ijomeh.1896.00657

[bibr_2] Bates B. Lennox A. Prentice A. Bates C. Page P. Nicholson S. & Swan G. (2014). *The National Diet and Nutrition Survey: Results from Years 1, 2, 3 and 4 (combined) of the Rolling Programme (2008/2009 – 2011/2012)*. TSO. https://assets.publishing.service.gov.uk/government/uploads/system/uploads/attachment_data/file/310995/NDNS_Y1_to_4_UK_report.pdf.

[bibr_3] Divakar U. Sathish T. Soljak M. Bajpai R. Dunleavy G. Visvalingam N. Nazeha N. Soh C. K. Christopoulos G. & Car J. (2019). Prevalence of Vitamin D deficiency and its associated work-related factors among indoor workers in a multi-ethnic Southeast Asian Country. *International Journal of Environmental Research and Public Health*, 17, 164. https://doi.org/10.3390/ijerph17010164. 31881679 10.3390/ijerph17010164 PMC6981433

[bibr_4] Eysenbach G. (2004). Improving the quality of web surveys: The Checklist for Reporting Results of Internet E-Surveys (CHERRIES). *Journal of Medical Internet Research*, 14(1), e8. 10.2196/jmir.6.3.e34 PMC1550605 15471760

[bibr_5] Fajardo V. C. de Oliveira F. L. P. Machado-Coelho G. L. L. Pimenta F. A. P. de Freitas S. N. Ribeiro A. L. P. Soares M. M. S. Lauria M. W. da Costa Farias R. França I. B. & do Nascimento Neto R. M. (2019). Effects of vitamin D supplementation on cardiovascular risk factors in shift workers. Study protocol for randomized, double-blind, placebo-controlled clinical trial. *Medicine*, 98, 18. 10.1097/MD.0000000000015417 PMC6504534 31045798

[bibr_6] Fitzpatrick T. (1988). The validity and practicality of sun-reactive skin types I through VI. *Archives of Dermatology*, 124, 869–871. 3377516 10.1001/archderm.124.6.869

[bibr_7] Holick M. (2019). *Vitamin D deficiency*. https://bestpractice.bmj.com/topics/en-gb/641/pdf/641/Vitamin%20D%20deficiency.pdf.

[bibr_8] Hourihane A. Glatt D. McClusky J. & Iniesta R. R. (2019). Vitamin D questionnaire validation and exploration of association with serum 25-hydroxyvitamin D in a UK adult population: A cross-sectional study and pilot study. *Endocrine Abstracts*, 65, P104. https://doi.org/10.1530/endoabs.65.P104.

[bibr_9] National Institute for Health and Care Excellence (NICE). (2018a). *Vitamin D deficiency in adults – treatment and prevention: Scenario: Management of vitamin D deficiency or insufficiency in adults*. https://cks.nice.org.uk/topics/vitamin-d-deficiency-in-adults-treatment-prevention/management/.

[bibr_10] National Institute for Health and Care Excellence (NICE). (2018b). *Vitamin D deficiency in adults – treatment and prevention*. https://cks.nice.org.uk/topics/vitamin-d-deficiency-in-adults-treatment-prevention/.

[bibr_11] O’Connor C. Glatt D. White L. & Iniesta R. R. (2018). Knowledge, attitudes and perceptions towards vitamin D in a UK adult population: A cross-sectional study. *International Journal of Environmental Research and Public Health*, 15, 2387. https://doi.org/10.3390/ijerph15112387. 30373274 10.3390/ijerph15112387 PMC6267199

[bibr_12] Ogrinc G. Davies L. Goodman D. Batalden P. Davidoff F. & Stevens D. (2015). *SQUIRE 2.0 (Standards for QUality Improvement Reporting Excellence): Revised publication guidelines from a detailed consensus process*. http://www.squire-statement.org/. 10.4037/ajcc2015455 26523003

[bibr_13] Palacios C. & Gonzalez L. (2014). Is vitamin D deficiency a major global public health problem? *Journal of Steroid Biochemistry and Molecular Biology*, 144, 138–145. 24239505 10.1016/j.jsbmb.2013.11.003 PMC4018438

[bibr_14] Roberts W. (2009). Skin type classification systems old and new. *Dermatological Clinics*, 27, 529–533. 10.1016/j.det.2009.08.006 19850202

[bibr_15] Scientific Advisory Committee on Nutrition (SACN). (2016). *SACN vitamin D and health report*. https://www.gov.uk/government/publications/sacn-vitamin-d-and-health-report.

[bibr_16] Sowah D. Fan X. Dennett L. Hagtvedt R. & Straube S. (2017). Vitamin D levels and deficiency with different occupations: A systematic review. *BMC Public Health*, 17, 519. https://doi.org/10.1186/s12889-017-4436-z. 28637448 10.1186/s12889-017-4436-z PMC5480134

